# Are Sleep Problems Related to Psychological Distress in Healthy Aging during the COVID-19 Pandemic? A Review

**DOI:** 10.3390/ijerph182010676

**Published:** 2021-10-12

**Authors:** Giuseppina Elena Cipriani, Massimo Bartoli, Martina Amanzio

**Affiliations:** Department of Psychology, University of Turin, 10124 Turin, Italy; giuseppinaelena.cipriani@unito.it (G.E.C.); martina.amanzio@unito.it (M.A.)

**Keywords:** sleep problems, healthy aging, psychological distress, depressive symptoms, anxiety, cognitive decline, SARS-CoV-2, COVID-19 pandemic

## Abstract

The SARS-CoV-2 pandemic, characterized by home confinement and other restrictive measures to reduce the spread of the infection, led to significant changes in people’s habits and lifestyle. One of the most common problems is the worsening of sleep quality or quantity, which could have negative effects on psychological wellbeing, particularly in older adults. The purposes of the present literature review considering healthy aging subjects are (a) to examine the existing research on sleep alterations during the current pandemic and (b) to highlight possible relationships between sleep problems and psychological distress. A systematic search strategy was implemented according to PRISMA guidelines in the international literature online databases, up to 1 July 2021. After identification and screening phases, 11 articles were included in this review. The studies found possible associations between sleep problems and mood changes—particularly in terms of depression and anxiety. In addition, altered sleep patterns seemed to be related to changes in individual aspects, lifestyle, and attitudes adopted by older adults during the COVID-19 lockdown. Thus, the pandemic could affect the sleep and psychological wellbeing of the older population, even in healthy aging.

## 1. Introduction

Aging is a heterogeneous process characterized by multiple biological, cognitive, emotional, and social changes. It can be considered in different ways according to the following adopted model: (1) “senescing”, in a biological perspective, with decreasing functionality and adaptability; (2) development, in a life course-oriented approach, with continuous changes and diverse stages throughout the entire life span [1].

In the biological view, aging can be defined as a decline in functional status, leading to inactivity, disabilities, and higher vulnerability to unfavorable outcomes [2,3]. Instead, in a life-span perspective, aging is portrayed as an economic and social resource. Thus, from this activity perspective derives the idea of “active aging”, which considers older adults in terms of participation in community life and politics and, above all, their rights to remain healthy [3]. Indeed, in the last few decades, a new concept of “healthy aging” has replaced the previous focus on active aging [4]. This new framework is concerned with persons aged 60 years and older [5] being and remaining a resource to their inner and outer systems. Older adults become actively involved in their wellbeing: meeting their needs, being independent and socially engaged, and living in environments suitable for their abilities and capacities. The aging process is the result of different influences in a functional and life-course approach. Primary importance is given to the functional ability, in which cognitive and physical capacities, environmental characteristics (factors forming the context of an individual), and their interactions are combined together [6]. Even the WHO report highlights various areas for primary intervention, unfortunately, sleep is not considered one of those.

Sleep is a biological function, an active behavior, crucial and vital for brain health and wellbeing at any age. It influences health status, quality of life, functioning, autonomy, and safety [7]. Indeed, recent findings suggest that good sleepers remain cognitively preserved for longer, report reduced rates of psychiatric diseases, and have a higher average life expectancy [8]. On the other hand, among individuals suffering from sleep disorders, such as insomnia, mood changes and cognitive dysfunctions can be observed [9], particularly in terms of depression [10,11] and anxiety [12], in attention, and memory [9].

The quality and quantity of sleep decreases with age [13]. Even though sleep quality does not have a unique definition [14], its assessment also considers quantitative aspects, such as those reported in the Pittsburgh Sleep Quality Index (PSQI) [15]. This instrument measures the following different sleep components: latency, duration, subjective sleep quality, efficiency, disturbance, daytime dysfunction, and use of hypnotic medication [15]. All these aspects together give an overall picture of the quality and patterns of sleep in adults. Sleep problems across the lifespan affect many biological processes leading to a significant reduction in individual wellbeing [16], possibly due to changes of brain homeostasis [8], which influences age-related neurophysiological processes and mood changes [17]. Moreover, mood disorders may exacerbate sleep disturbances in older adults [18]. On the other hand, sleep problems (i.e., insufficient sleep duration) may contribute to the manifestation of mood changes through both hypothalamic-pituitary-adrenal (HPA) axis misregulation, and excessive activation of the sympathetic nervous system [19]. In this direction, poor sleep is a common manifestation of both depression and anxiety [20], as they may influence circadian disruption [21]. Not surprisingly, sleep problems are considered symptoms of anxiety and depression [22].

Several studies have shown bidirectional associations between sleep problems and mood changes, in terms of anxiety and depression [23,24,25,26], attesting the impact of psychological distress. Although the etiological relationship between these problems is still unclear [27], some hypotheses on their reciprocal influence have been made, such as the sharing of common risk factors [28]—i.e., genetic, familial, social, or environmental—and the presence of a common neurobiological substrates [27].

Psychological distress is defined as a set of non-specific symptoms concerning depressive mood and anxiety manifestation [29,30]. In fact, many self-report instruments for depression and anxiety assess psychological distress [31]. It refers to a state of emotional suffering associated with stressors that are difficult to deal with in daily life [32]. Although these kinds of mood fluctuations are considered as normal in most people, psychological distress may be a precursor of a variety of clinical conditions, such as anxiety and major depressive disorders, characterized by somatization forms [31]. In addition, psychological distress may influence sleep patterns [33]. In particular, some authors found an association between sleep duration and psychological distress [19,34]. Various kinds of insomnia seem to be related to different mood changes: in fact, onset insomnia was associated with anxiety, while maintenance insomnia was related to depressive symptoms in older adults [35]. Furthermore, psychological distress seems to exacerbate age-related sleep problems [24]. In particular, a reduced sleep length and continuity, a decline in sleep depth, a decreased REM sleep latency, and insomnia are more severe in older adults with depressive symptoms [36,37]. Conversely, sleep onset difficulties are more common in older adults with anxiety [38].

Regarding the relationship between sleep disorders and cognitive dysfunctions, previous studies showed that poor sleep is related to worse cognitive functioning in older people (e.g., [39]). For example, some studies show an association between daytime sleepiness and impaired attention, orientation, memory, and slow processing speed [40,41,42]. Sleep problems are also associated with subjective cognitive decline [43] and cognitive impairment [44]. Nevertheless, this relationship is bidirectional: sleep problems could affect cognitive decline but also sleep disruption could be an early sign of cognitive impairment [42].

Aging is also characterized by a reduced proportion of Rapid Eye Movement (REM) sleep, a substantial reduction in non-REM (NREM) stage three sleep, and fragmented sleep with frequent interruptions, leading to a shorter total sleep time and greater amount of wake after sleep onset [45]. This leads older people to a reduction in daily functioning [46], increasing the risk of developing cognitive decline [47] and highlighting a potential causal association between sleep disorders and the pathogenesis of neurocognitive diseases, as typical aspects of neurodegenerative disorders [8]. 

The above aspects and their associations are more emphasized during periods of extreme hardship, such as the COVID-19 pandemic, characterized by home confinement and other restrictive measures to reduce the spread of the infection [48]. In the last year, many studies have shown the lockdown effects experienced by people of different ages. In Italy, for instance, the SARS-CoV-2 pandemic has undermined psychological wellbeing: isolation, fear of being infected by the virus, and feelings of frustration, anxiety, boredom, and uncertainty have affected daily functioning and sleep [49]. Social distancing and isolation can lead to depressive symptoms by exacerbating worries and loneliness, leading to more negative outcomes related to the COVID-19 lockdown [50]. Furthermore, restrictive measures may also have a negative impact on the cognitive functioning of older adults [51].

These problems are also found in healthy aging subjects. In particular, a recent study found that the so-called "lockdown fatigue" seemed to be related to the complex interrelationship between cognitive, psychological, and physical factors in the older population [52]. In this direction, another study showed that mood deflection, in terms of anxiety, a physical pre-frailty status [53], and a decrease in the speed of processing environmental information, could influence the perceived threat of SARS-CoV-2 infection risk in healthy aging subjects [54].

A recent review has focused on the impact of the COVID-19 pandemic by considering sleep disturbances in the older population [21]. The authors found that sleep deprivation and fragmentation, and obstructive sleep apnea may cause a decline of immune responses, leading older individuals to be more likely to contract an infection and, furthermore, a worse prognosis. However, they have not investigated the relationship between sleep problems and psychological distress in healthy aging subjects in depth. Another recent review [55] found that insomnia was the most common sleep disorder in older adults. The authors hypothesized that this sleep disturbance may increase during the second wave of the SARS-CoV-2 pandemic. Nevertheless, they particularly focused on older patients with chronic diseases (i.e., cardiovascular disease and psychiatric disorders, pulmonary disease and neurological disorders, diabetes, and cancer), which may directly impact sleep misregulation [56].

To the best of our knowledge, our review is the first conducted to synthesize the existing literature on sleep problems and psychological distress in healthy aging in relation to the SARS-CoV-2 pandemic.

To this end, we selected studies from the literature that allowed us to investigate and summarize (a) the type and frequency of sleep disturbances; (b) possible associations between sleep disturbances and other variables related to the COVID-19 lockdown, such as negative changes in psychological wellbeing, in older adults during the COVID-19 pandemic.

## 2. Materials and Methods

### 2.1. Search Strategy 

To identify studies on sleep disturbances in the older population during the COVID-19 pandemic, a systematic search strategy was implemented in the international literature online database (MEDLINE database with PubMed literature search). We entered the following query terms: (*Aging*) AND (*Sleep*) AND (*COVID-19*), searching for relevant scientific literature published up to 1 July 2021. 

In addition, a second systematic strategy was implemented in the international literature online databases (ProQuest and Scopus), in order to further investigate the relationship between sleep problems and mood changes. We entered the following query terms: (*Aging*) AND (*Sleep*) AND (*COVID-19*) AND (*Psychological Distress*), searching for relevant scientific literature published up to 1 July 2021.

This review was conducted adopting the guidelines of the Preferred Reporting Items for Systematic Review and Meta-Analyses (PRISMA) [57], adapted to our investigation.

Two authors (M.B. and G.E.C.) carried out the study selection process independently. They identified articles by title, abstract, and full text. Any disagreements were discussed and resolved. Finally, one author (M.A.) supervised this phase.

### 2.2. Inclusion and Exclusion Criteria

To guarantee the selection of pertinent articles, we included only studies (a) on sleep problems in healthy aging older adults and (b) during the SARS-CoV-2 pandemic. Moreover, we considered studies (c) on sleep disturbances in cognitively impaired vs. healthy aging subjects—indeed, studies that only analyzed subjects with neurocognitive disorders were not considered; or (d) comparing older (60 years and older) and younger subjects. Finally, we excluded (a) sleep disorders reported in COVID-19 patients, characterized by sleep difficulties [58]; (b) sleep problems regarding any health conditions that could influence sleep quality or quantity; (c) studies on sleep problems that focused only on individuals with clinical conditions affecting healthy aging (e.g., neurocognitive disorders).

### 2.3. Study Selection

After discussing the eligibility criteria for each study, two authors (G.E.C. and M.B.) independently analyzed the following information: population and its characteristics, sample size, procedures, study design, and outcomes. In case of disagreement, a consensus was reached or the judgment of a third author was sought (M.A.)

### 2.4. Outcome Measures

This review was carried out to find information on sleep disturbances in normal cognitive aging subjects during the SARS-CoV-2 pandemic. The following information was also examined in relation to sleep issue: (a) cognitive functioning; (b) psycho-geriatric factors.

## 3. Results

### 3.1. Study Selection

**Figure 1:**
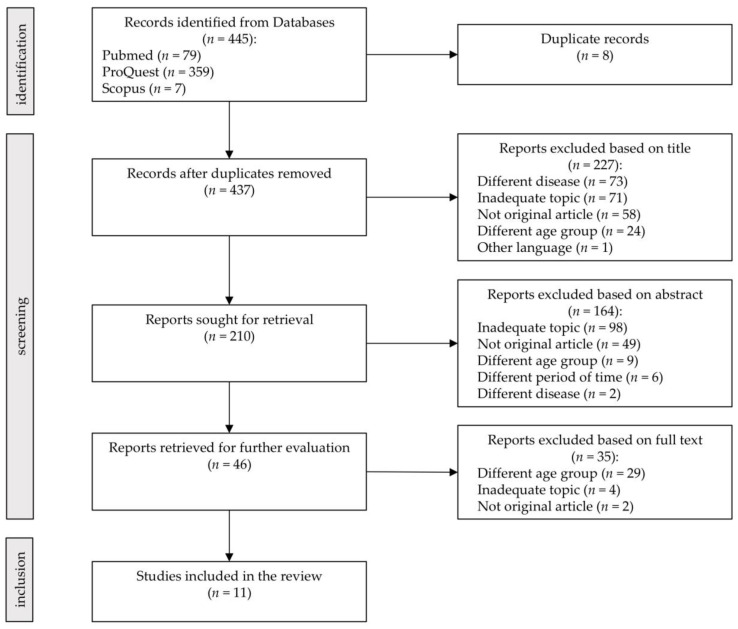
Study selection. The PRISMA 2020 statement [57].

The initial literature search process led to 445 articles meeting our eligibility criteria. Of these, eight were removed before screening, as duplicates. After the identification and screening phases, 11 studies [59,60,61,62,63,64,65,66,67,68,69] were included and 426 were excluded (See Flowchart, Figure 1). The number and reasons for the exclusion of the articles were the following:

- 109 were not original articles (43 reviews, 24 abstracts, 12 perspectives, 9 study protocols, 6 letters, 5 commentary, 3 pre-prints, 2 case reports, 2 editorials, 1 supplement, 1 opinion, and 1 corrigendum);
- 75 referred to subjects not experiencing healthy aging (different diseases): people with health conditions that could influence sleep quality or quantity, such as in COVID-19 patients;
- 62 did not take into consideration older adults (different age group);
- 173 were related to inadequate topics (studies focusing on issues not concerning the aims of our review);
- 7 failed to meet some inclusion criteria (six did not refer to sleep problems during the SARS-CoV-2 pandemic, one was not in English).

### 3.2. Description of the Selected Studies

The characteristics of the selected articles are reported in Table 1.

To analyze the impact of the home confinement, Costi and colleagues [59] administered an online survey from May to June 2020. Among 1826 Italian participants between the ages of 18 and 70, 194 were 65 years and older. The survey collected information on sleep quality, lifestyle behaviors and changes (diet, physical activity, smoking, and use of alcohol), and symptoms of psychological distress (i.e., fear, loneliness, tension, uncertainty, upset, and worry) during and immediately after the lockdown period. In the general sample, more than half the participants reported sleep changes in terms of reduced hours or disruption of sleep during the lockdown period. The results showed that sleep changes were negatively influenced by some health determinants: symptoms of psychological distress (loneliness: OR 3.27; 95% CI 2.23–4.79 and tension: OR 3.88; 95% CI 2.74–5.52); diet and physical activity (OR 4.19; 95% CI 2.51–6.96 and OR 1.68; 95% CI 1.18–2.40, respectively); and financial problems (some: OR 1.86; 95% CI 1.27–2.72, many: OR 7.27; 95% CI 3.59–14.73). In particular, regarding the aged-adult group, almost 30% reported sleep changes, in terms of poor sleep quality, significantly associated with feelings of uncertainty (*p* = 0.040).

Two studies [60,61] highlighted a relationship between sleep disturbances and mood changes, also considering possible associations between sleep patterns and cognitive functioning in older adults during the COVID-19 pandemic. 

Carlos et al. [60] analyzed the lockdown effects in a sample of 204 Italian older adults (≥65 years old), stratified by their cognitive status. Specifically, the subjects were divided into the following three groups following their baseline cognitive state: normal old (NOLD: *n* = 162), mild Neurocognitive Disorder (Mild-NCD: *n* = 24), and major Neurocognitive Disorder (Major-NCD: *n* = 18). This cross-sectional telephonic survey was conducted between April and May 2020, before the Italian “phase 2”, when the restrictive measures started to be eased. The authors found that almost half of the sample (48.5%) reported sleep problems, in terms of difficulty in falling asleep, frequent arousals, early morning awakening, and nightmares. Of these, more than 80% pre-existed in the Major-NCD and NOLD groups, which did not show great changes during the lockdown. Conversely, subjects with Mild-NCD reported new-onset sleep disturbances. Furthermore, logistic regression analyses showed that poor sleep and general health issues independently were more likely to exacerbate depressive symptoms (OR 2.29, CI 1.06–4.93, *p* = 0.034 and OR 2.45, CI 1.16–5.16, *p* = 0.018, respectively), and that new-onset sleeping problems strongly increase the risk of subjective memory complaints (OR 10.26, CI 1.13–93.41, *p* = 0.039).

In the second study, De Pue and colleagues [61] examined the impact of the COVID-19 period on the older population (mean age = 73 years, SD = 6.99), characterized by high socioeconomic status and good health condition, living in Flanders (Belgium). Six hundred and forty subjects (377 females) responded to an online survey lasted from May to June 2020. They were assessed on changes related to sleep quality, perceived wellbeing, activity level, and cognitive functioning, through self-report measures. Regarding sleep, the participants were asked to reply on an 11-point scale ranging from very poor to very good quality of sleep. Of the sample, 76% reported decreased levels of subjective wellbeing, included poor sleep. Specifically, during the lockdown, sleep quality was significantly worse than during the pre-pandemic period (*t*(639) = −5.87, *p* < 0.001, d = 0.23). A small group of participants reported changes in their cognitive functioning. They indicated more problems in remembering, concentration, multitasking, recalling, and forgetfulness (in 8, 12, 6, 10, and 10%, respectively). Furthermore, linear regression analysis showed an association between sleep changes occurred during the pandemic period and depressive symptoms. Indeed, reduced sleep quality was associated with gender (being male) and more depressive symptoms (CI 0.055–0.45, *t*(598) = 2.52, *p* < 0.02; CI −0.16–0.084, *t*(605) = −6.60, *p* < 0.001, respectively). On the other hand, no association was found between sleep disturbances and cognitive functioning.

Two studies [62,63] assessed participants’ functionality, in terms of physical activity, frailty status, and autonomy to perform basic or instrumental daily living activities, during the COVID-19 lockdown period. 

In the first study, to investigate changes in healthy behaviors and lifestyle during the COVID-19 pandemic, Zach et al. [62] administered an online survey from April to May 2020 in Israel. Participants, categorized in three different age groups (45–59, 60–69, ≥70), were asked to reply to different items on physical activity, psychological wellbeing, diet, and other behaviors—sleep included—before and during the pandemic. According to their level of physical activity, the subjects were categorized as inactive, insufficiently active, and sufficiently active. Considering sleeping time, results showed a positive association with physical inactivity (significant differences and interaction between age groups: F (2; 1168) = 6.490, *p* < 0.01 and F (6; 1168) = 3.132, *p* < 0.01, respectively). In particular, in the oldest group (≥70), inactive subjects slept more compared with others; furthermore, people aged 60–69, active both before and during the home confinement, reported more sleeping hours. As for psychological aspects, younger subjects reported more depressive symptoms compared to the older participants; nevertheless, in the 60–69 age group, the not physically active subjects showed more depressive symptoms than those that were active (*p* < 0.001).

In the second study, Wang and colleagues [63] assessed the influence of frailty status and polypathology in psychological distress changes before and during the COVID-19 pandemic in Chinese people 60 years and older (median age: 70 years). A total of 2785 community-dwelling participants, included in the Shandong Rural Elderly Health Cohort study, responded to a face-to-face interview conducted from August to September 2020. The subjects answered to different aspects on lifestyle behaviors, sleep quality included, and psychological distress (such as anxiety and depression). Furthermore, they underwent a frailty and physical mobility assessment. To assess sleep quality, the authors used the Pittsburgh Sleep Quality Index (PSQI) [15], which measures all the characteristics of sleep. Comparing before with during the pandemic, the percentage of the sample reporting good sleep decreased from 46 to 36%; instead, the percentage of participants sleeping poorly increased from 54 to 64%. Even though the results showed these differences, no significances were reported. In general, the respondents showed increased levels of psychological distress during the pandemic period associated with both frailty progression (*p* < 0.001) and polypathology (*p* < 0.001).

Other studies [64,65,66,67] considered individual aspects, lifestyle, and attitudes adopted by older adults during the COVID-19 lockdown period. 

Bann and collaborators [64] evaluated changes in health status and behaviors before and during the COVID-19 lockdown in the United Kingdom. An online survey collected data from participants previously included in five birth cohort studies, among which were those born in 1946 and 1958, aged 74 and 62, respectively. Results from logistic regression analysis showed changes in behaviors occurred during the home confinement, with older respondents reporting a lower impact compared to younger counterparts. Regarding sleep, there was a positive correlation before and during lockdown (Spearman’s R = 0.55). In addition, all cohorts differed from previous habits, either with increased or decreased amounts of sleep. Sleeping less was reported by 10 and 10.8% of the older subjects before the pandemic and the percentages increased to 15.6 and 16.1% in the same groups during the COVID-19 outbreak. 

Emerson [65] studied the impact of social distancing due to COVID-19 restrictive measures in 883 American older adults. A web-based survey collected information from people 60 years and older between March and April 2020. The items focused on social contacts, loneliness, distress (in terms of anxiety), and behavioral and communication changes (e.g., sleeping, smoking, drinking, eating, and physical activity). The results showed that about one third of the sample reported sleep changes during the COVID-19 lockdown. In particular, 27.1% slept more than usual, while 15.8% slept less. When considering their age, the younger group (60–70 years old) reported sleeping less compared with the older group (≥71) (*p* = 0.010). Furthermore, 42.9% of the respondents felt lonely some or most days (42.5 and 43.5% in the younger and older groups, respectively) and 36.9% reported moderate or high levels of distress; in particular, the older subjects (aged 71+) reported to be less stressed than the younger group (60–70 years) (*p* = 0.000). To summarize, unlike their older counterparts, the younger subjects (aged 60–70 years old) reported less healthy behaviors: eating more, sleeping less, being more stressed, and drinking more alcohol, even thought they were more physically active. 

Grossmann et al. [66] investigated the role that worries and resilience play in modulating loneliness and sleep problems related to the COVID-19 pandemic in older adults. During the Israeli lockdown, between March and April 2020, 243 subjects (mean age = 69.76, SD = 6.69), with a medium–high socioeconomic status and a self-reported good health condition, replied to an online questionnaire that measured medical conditions, behavior changes, and distress factors—such as loneliness and worries—related to SARS-CoV-2, resilience, and sleep problems. The respondents were asked about satisfaction with current sleep, difficulties in falling asleep, and feelings of tiredness or low energy since the outbreak of the pandemic. The authors found these sleep issues were positively associated with distress factors, such as loneliness and worries, and negatively related to resilience. Results from regression analysis showed that a higher level of COVID-19-related loneliness was associated with more sleep disturbances (*p* < 0.001). Furthermore, such association was stronger in older subjects more worried and less resilient compared with older adults who were less worried and more resilient (*p* < 0.05). 

Finally, to analyze the perception of changes in habits and behaviors during the COVID-19 confinement, Machado-Lima and colleagues [67] questioned younger and older subjects in Brazil between May and June 2020. Among the participants, 139 were young adults (mean age = 43.15; SD = 10.92); 437 were older adults (mean age = 67.59; SD = 6.13). In this cross-sectional study, the sample was also asked to respond to items on sleep timing and quality, emotional status, health, physical activity, and social contacts both before and during the COVID-19 lockdown. All the participants reported changes in their routine (95 and 96.8% in the younger and older group, respectively). The online survey results highlighted more tiredness, lower levels of physical activity, and more sleep disturbances, both in the younger and older adults. Particularly, both age groups showed a decreased sleep quality during the home confinement comparing with the pre-pandemic era (*p* < 0.0001 for adult and older adult comparisons). Furthermore, the older group showed more sleep problems and difficulty in carrying out daily activities at home compared with the younger group. On the other hand, the younger and older adults did not differ on mood alternations. Specifically, both of them indicated an intermediate status of happiness (vs. sadness) and excitement (vs. discouragement).

Inconsistent with the results described previously, some studies [68,69] found little or no significant differences with respect to sleep patterns during the COVID-19 and pre-pandemic period. 

Arai and colleagues [68] conducted a cross-sectional telephonic survey on 487 Japanese older people (median age: 89.3), without limitations in instrumental activities of daily living nor cognitive impairment. The subjects, included in a longitudinal cohort study—the Kawasaki Aging and Wellbeing Project, were previously assessed on their cognitive, functional, and psychological status. Between May and August 2020, the participants were asked to reply to 12 items on behaviors and habits during the pandemic period. Almost the entire sample (94.5%) reported no changes in basic lifestyle behaviors regarding sleeping (in general terms), eating, drinking, and smoking. 

Topriceanu and colleagues [69] investigated possible associations between being a key worker and several health and behavioral outcomes during the COVID-19 lockdown. The authors implemented an online survey, which collected data from four British birth cohorts. Among 13,736 participants, 5119 were from the National Child Development Study, born in 1958 and aged 62 in 2020. During the home confinement, British key workers were at a higher risk of being infected with SARS-CoV-2 and experiencing conflicts; in addition, they were less likely to have financial problems or abuse smoking and alcohol. Regarding sleep duration, only 15% of the older adults group slept less during the lockdown period. Nevertheless, being a key worker was not related to sleep deprivation nor psychological distress in any of the four cohorts (pooled OR 1.06, 95% CI 0.94–1.19, *p* = 0.350 and OR 0.95, 95% CI 0.85–1.05, *p* = 0.320, respectively).

**Table 1:** Characteristics of the 11 studies included in the review.

Reference	Setting	Period (2020)	Country	Participants (*n*)	Females/Males (%)	Age in Years (M ± SD)	Sleep Assessment	Cognitive Assessment	Psychological Assessment	Geriatric Assessment
Arai et al., 2021 [68]	telephonic survey	May–August	Japan	487	50.5/49.5	≥85; range: 85–89	item on sleep changes *			
Bann et al., 2021 [64]	online questionnaire	May	United Kingdom	5654	49.35/50.65	62 and 74	item on sleep duration *			
Carlos et al., 2021 [60]	telephonic survey	April–May	Italy	162	57.4/42.6	≥65; range: 75.3–84	items on sleep problems *	items on subjective memory complaints	depression (GDS-5) [70,71]	
Costi et al., 2021 [59]	online survey	May–June	Italy	194	N.A.	≥65	items on sleep quality *		item on psychological distress	
De Pue et al., 2021 [61]	online survey	May–June	Belgium	640	58.99/41.00	≥65 (73 ± 6.99)	item on sleep quality *	subjective cognitive functioning (CFQ [72,73]); items on subjective cognitive change	depression (GDS-15 [74,75]); wellbeing (PWI-A [76,77]); resilience (BRS [78,79])	
Emerson, 2020 [65]	online survey	March–April	United States	833	80.5/19.5	≥60; range: 60–85+	items on sleep duration *		items on loneliness and stress	
Grossman et al., 2021 [66]	online questionnaire	March–April	Israel	243	69.1/30.9	≥60 (69.76 ± 6.69)	adapted items on sleep quality and changes (Insomnia severity index [80]; the PHQ-9 depression questionnaire [81])		loneliness (UCLA Three-Item Loneliness Scale [82]); resilience (CD-RISC [83])	
Machado-Lima et al., 2021 [67]	online questionnaire	May–June	Brazil	437	68.6/31.4	≥60 (67.59 ± 6.13)	item on sleep quality *		items on emotional status	
Topriceanu et al., 2021 [69]	online survey	May	United Kingdom	5119	52.49/47.51	62	item on sleep duration *		items on psychological distress	
Wang et al., 2021 [63]	face-to-face survey	August–September	China	2785	63.55/36.45	≥60; median age: 70	PSQI [15]		psychological distress (Kessler Psychological Distress Scale [32,84])	frailty; ADL [85]
Zach et al., 2021 [62]	online questionnaire	April–May	Israel	557	64.27/35.56	≥60; range: 60–90	item on sleep duration *		resilience (the Connor-Davidson Resilience Scale); depression (Kandel and Davies’ questionnaire [86])	

Note: *n* = number. M = mean. SD = standard deviation. GDS = Geriatric Depression Scale. N.A. = Not Available. CFQ = Cognitive Failures Questionnaire. PWI-A = Personal Wellbeing Index-Adults. BRS = Brief Resilience Scale. PHQ = Patient Health Questionnaire. UCLA = University of California, Los Angeles. CD-RISC = Connor-Davidson Resilience scale. PSQI = Pittsburgh Sleep Quality Index. ADL = activities of daily living. * Sleep data collected from ad hoc survey items.

## 4. Discussion

In our review, despite the fact a few selected studies found none or little differences on sleep habits during the current pandemic period [68,69], the others observed that the older population, also considering subjects in the healthy aging group, was prone to experience sleep disturbances during the COVID-19 confinement [59,60,61,62,63,64,65,66,67]. In particular, some of the selected studies (4 out of 11) took into consideration the possible association between sleep disturbances and mood changes in the older population during the lockdown period [59,60,61,66]. All of them found a relationship between sleep problems and psychological distress. In particular, depression seems to be negatively related to older adults’ sleep during the COVID-19 pandemic [60,61]. Furthermore, loneliness, which refers to psychological distress, seems to be associated with sleep problems particularly in aged people with more worries about SARS-CoV-2 [66]. In addition, feelings of uncertainty, which are connected with anxiety-related disorders [87], may be related to poor quality of sleep [59]. 

Indeed, during a difficult period, such as the COVID-19 pandemic, social distancing and isolation might exacerbate sleep problems and psychological distress already present in the older population, leading to greater negative health status outcomes [50]. Moreover, cognitive, psychological, and functional aspects in older adults in healthy aging may have a critical role in both fatigue due to home confinement restrictive measures [52] and the perceived threat of SARS-CoV-2’s risk of contagion [54].

Although the cross-sectional nature of the data does not allow us to infer with whether mood changes are precursors to sleep-related issues during the COVID-19 period or whether the lockdown triggered or intensified such psychological aspects, these findings suggest that depression and anxiety seem to be related to sleep disturbances in older adults during the SARS-CoV-2 pandemic. Furthermore, some studies have analyzed other aspects related to psychological changes in wellbeing, such as the level of stress and loneliness [63,65,66,67,69], which may affect the manifestation of anxiety [88] and depressive symptoms [89], respectively.

Only two studies analyzed the association between sleep disturbances and cognitive functioning [60,61]. In particular, Carlos et al. [60] found an association between cognitive functioning and sleep issues, comparing normal old (79% of the sample) with patients with mild (12%) and major NCD (9%). In fact, although the presence of sleep problems alone did not correlate with subjective memory changes, new-onset sleep disturbances strongly increased their likelihood. 

Conversely, De Pue and colleagues [61] found no association between sleep disturbances and self-reported cognitive complaints during the pandemic period in older subjects. However, these results may have been affected by the fact that cognitive data were collected through self-reported questionnaires and not by a performance-based assessment.

Finally, only one study assessed subjects’ independence of daily living and frailty status. Wang and colleagues [63] assessed participants’ frailty status (adopting the phenotypic model [53]) and limitations in basic activities of daily living (measured with an ADL score [85]). Their results showed increased levels of psychological distress and sleep problems during the pandemic period associated with both frailty progression and polypathology.

Sleep quality is important for our mental health, in fact, and sleep disorders are associated with psychopathology [49], such as alcohol abuse, depression, and anxiety disorders [90]. Stressful events influence sleep patterns, particularly among vulnerable people, such as the older adults. Indeed, the COVID-19 pandemic affected sleep quality and duration [91]. Aging is characterized by progressive sleep and other circadian rhythms alterations [92] involved in the regulation of most biological systems [93]. Thus, sleep disturbances are frequent in older people [94] and have become even more common during the COVID-19 lockdown [50,91]. In addition to the greater psychological distress reported in different ages [49], older adults can be also characterized by a tendency toward impaired circadian alignment [95].

In this review, our results on sleep changes are not homogeneous. As previously stated, some studies showed associations between sleep problems and mood changes; other studies considered the impact of individual aspects, lifestyle, and attitudes adopted by older adults during the COVID-19 lockdown; finally, others found no significant differences in sleep patterns comparing before with during the pandemic period. In our recent study [52], which aimed to analyze the influence of cognitive, psychological, and functional factors on the lockdown fatigue, we also asked a group of healthy aging subjects if they had experienced any lifestyle changes, such as in sleep quality, during the COVID-19 home confinement. Consistent with the evidence in the literature, we observed heterogeneous results (i.e., improvement, similarity, and worsening, in 12, 56, and 32% of the sample, respectively). 

The reason for this variety may lay in the different methodological approaches adopted by the researchers. The COVID-19 pandemic was a peculiar period in which even conducting scientific research was a challenge. Different age groups and convenience samples, but also telephone or online surveys, were used to investigate variables of interest. For example, in the majority of the selected articles, data on sleep were collected with ad hoc or adapted items, and only in one study [63] with a specific tool, valid and reliable in the older population, such as the Pittsburgh Sleep Quality Index (PSQI) [15]. Thus, most authors administered only one item on a peculiar aspect of sleep (i.e., quality, duration, interruptions, problems, or changes) during the COVID-19 lockdown [61,62,64,67,68,69].

Conversely, Wang and colleagues [63] focused on all the following sleep components: latency, duration, subjective sleep quality, efficiency, disturbance, daytime dysfunction, and use of hypnotic medication. Interestingly, their results showed sleep problems and psychological outcomes during the pandemic period. Specifically, they found both poorer sleep quality and greater psychological distress, associated with frailty progression and polypathology, during the pandemic period.

Many studies have shown a significant relationship between sleep disturbances and mood changes in older adults, particularly in terms of depression and anxiety [18]. Specifically, they both seem to be related to different sleep problems, such as poor quality of sleep [96,97,98], increased sleep onset latency [38], and daytime sleepiness [99]. Furthermore, anxiety and depression are the most frequently reported and studied psychological changes in the literature regarding the COVID-19 pandemic [100]. They are very common in older adults, as a consequence to social isolation [101], particularly with regard to fear of contagion [54,102] and the so-called “lockdown fatigue” [52]. 

Although social isolation is the most effective measure to safeguard against COVID-19, it exposes individuals to physical and mental health issues [103], such as changes in sleep patterns. The prevalence of sleep disturbances is more common in older adults compared with younger subjects [94,104]. Furthermore, home confinement, social isolation, and physical inactivity may exacerbate these kinds of problems in the older population [105].

To summarize, even if our results are heterogeneous, the majority of the selected studies show a worsening in sleep patterns, which seems to be correlated with psychological and physical changes in older adults.

## 5. Limitations of the Study and Future Research

The present review represents the first attempt to describe and synthetize the data on sleep patterns and psychological related factors in older adults during the COVID-19 pandemic. Some limitations should be addressed, and the results considered with caution.

First, the analyzed studies used various instruments for the assessment of sleep problems, also focusing on different features (e.g., quality, duration, disturbances). This aspect did not allow us to carry out a systematic review, nor a meta-analysis, on the collected data. Furthermore, most of the studies adopted ad hoc survey items instead of specific tools for the sleep assessment. Such aspects may have led to bias regarding the assessment of the actual sleep problems experienced by older subjects included in the studies. However, the challenge in conducting a more in-depth sleep evaluation (i.e., face-to-face assessment) may be due to home-confinement measures adopted to limit the spread of SARS-CoV-2. Nevertheless, the paucity of the literature on this topic on the one hand, and the importance of this issue on the other, make more analysis urgent. Indeed, most of the selected studies showed a worsening in sleep quality and/or quantity in older adults during the COVID-19 pandemic.

Second, nearly all the studies administered online surveys. It should be noted that older people might experience technology disadvantages. Furthermore, almost none of the selected studies recruited a representative sample of the older population. Thus, this approach makes the results not fully generalizable.

Third, most of the studies adopted a cross-sectional design, which does not allow for inferring the direction of associations. 

Finally, regarding the neuro-psycho-geriatric assessment, some critical issues should be considered. The majority of the studies did not perform a cognitive assessment to verify whether the subjects were in normal cognitive aging; this aspect could have led to biased results. Considering the close relationship between sleep disorders and cognitive decline in the older population [47], it would be useful to investigate this aspect during the pandemic period through a more in-depth neuropsychological assessment. In addition, other factors that may affect cognitive decline should be considered, such as depression and anxiety [106,107], which have been previously related to sleep disorders. In fact, during the COVID-19 lockdown, many people experienced feelings of frustration, boredom, but also depression and anxiety [49]. This condition influenced lifestyle habits and behaviors with consequences on sleep quality and daily functioning [108]. 

The lifestyle changes occurred because of the COVID-19 home confinement, which might have exacerbated symptoms in older adults, particularly the frailest ones, leading to the so-called “Corona-Frailty” [109] and to higher perceived threat of being infected by the COVID-19 virus [54]. Indeed, frailty is a condition characterized by greater vulnerability associated with age-related decline in biological functions and systems, which might affect the ability to deal with stressors [6]. In the literature, little is known about older people’s functionality during the COVID-19 lockdown. Indeed, frailty could not be fully investigated without a face-to-face assessment. Furthermore, studies conducted before the COVID-19 pandemic highlighted correlations between mental health and both polypathology [110,111] and frailty status [112,113]. Future studies should investigate these relationships in the older population considering home confinement measures due to COVID-19. 

To summarize, different age ranges, gender, education, health status, lockdown period and restrictions, sample size and recruitment, but also sleep, cognitive, psychological, and functional assessments could have influenced the results.

## 6. Conclusions

The evidence from the selected studies seems to suggest that sleep problems may affect psychological and physical wellbeing in older people during the COVID-19 pandemic [59,60,61,66], although the results are not definitive due to the little evidence available on healthy aging.

Since the studies reviewed were conducted in the first part of the COVID-19 pandemic (between March and September 2020), future research is needed to assess the long-term effects of SARS-CoV-2 restrictive measures using a homogeneous methodology to assess sleep disturbances and its consequences on mental and physical health in older adults.

These aspects are important to consider as changes in sleep patterns, sleep architecture, and circadian rhythm might influence biological systems involved in age-related and chronic diseases [17]. Nevertheless, definitive conclusions cannot also be made due to the reciprocal relationship between sleep and mood changes, which should be better clarified by new research studies. However, a better comprehension of age-related sleep changes may help us improve our knowledge to develop new solutions for healthcare approaches in the older population.

## Data Availability

The protocol of the present Review can be available on request from the corresponding author for valid and important reasons. The present Review was not registered in any International Database.
